# Cerebral microbleeds in a neonatal rat model

**DOI:** 10.1371/journal.pone.0171163

**Published:** 2017-02-03

**Authors:** Brianna Carusillo Theriault, Seung Kyoon Woo, Jason K. Karimy, Kaspar Keledjian, Jesse A. Stokum, Amrita Sarkar, Turhan Coksaygan, Svetlana Ivanova, Volodymyr Gerzanich, J. Marc Simard

**Affiliations:** 1 Department of Neurosurgery, University of Maryland School of Medicine, Baltimore, Maryland, United States of America; 2 Department of Pathology, University of Maryland School of Medicine, Baltimore, Maryland, United States of America; 3 Department of Physiology, University of Maryland School of Medicine, Baltimore, Maryland, United States of America; National University of Singapore, SINGAPORE

## Abstract

**Background:**

In adult humans, cerebral microbleeds play important roles in neurodegenerative diseases but in neonates, the consequences of cerebral microbleeds are unknown. In rats, a single pro-angiogenic stimulus *in utero* predisposes to cerebral microbleeds after birth at term, a time when late oligodendrocyte progenitors (pre-oligodendrocytes) dominate in the rat brain. We hypothesized that two independent pro-angiogenic stimuli *in utero* would be associated with a high likelihood of perinatal microbleeds that would be severely damaging to white matter.

**Methods:**

Pregnant Wistar rats were subjected to intrauterine ischemia (IUI) and low-dose maternal lipopolysaccharide (mLPS) at embryonic day (E) 19. Pups were born vaginally or abdominally at E21-22. Brains were evaluated for angiogenic markers, microhemorrhages, myelination and axonal development. Neurological function was assessed out to 6 weeks.

**Results:**

mRNA (*Vegf*, *Cd31*, *Mmp2*, *Mmp9*, *Timp1*, *Timp2*) and protein (CD31, MMP2, MMP9) for angiogenic markers, *in situ* proteolytic activity, and collagen IV immunoreactivity were altered, consistent with an angiogenic response. Vaginally delivered pups exposed to prenatal IUI+mLPS had spontaneous cerebral microbleeds, abnormal neurological function, and dysmorphic, hypomyelinated white matter and axonopathy. Pups exposed to the same pro-angiogenic stimuli *in utero* but delivered abdominally had minimal cerebral microbleeds, preserved myelination and axonal development, and neurological function similar to naïve controls.

**Conclusions:**

In rats, pro-angiogenic stimuli *in utero* can predispose to vascular fragility and lead to cerebral microbleeds. The study of microbleeds in the neonatal rat brain at full gestation may give insights into the consequences of microbleeds in human preterm infants during critical periods of white matter development.

## Background

In adult humans, “cerebral microbleeds” (brain microbleeds, microhemorrhages, punctate hemorrhages) are increasingly diagnosed using susceptibility-weighted magnetic resonance imaging (MRI), are frequently associated with white matter abnormalities (leukoaraiosis), and are increasingly recognized to play important roles in several neurodegenerative diseases [1–5].

By contrast, in neonates, cerebral microbleeds are not widely recognized, and available studies on this pathological entity are extremely limited. In MRI case series, evidence of prior hemorrhage (hemosiderin), varying from isolated punctate lesions to more extensive hemorrhages, have been reported in 40–60% of preterm infants [6,7]. In a cohort of infants who underwent surgery for congenital heart disease, cerebral microbleeds were associated with significant neurodevelopmental impairment [8].

In rodents, hemorrhages in the neonatal brain are more harmful than in the adult brain [9]. Haptoglobin, an acute phase protein that is required for scavenging blood breakdown products [10], is nearly absent at birth [11]. Thus, blood and blood breakdown products that are present at birth can persist for many days, and thus have a protracted opportunity to induce oxidative damage. Excess periventricular iron, which can arise from hypoxia/ischemia (H/I) or from microbleeds, is strongly linked to oligodendrocyte apoptosis and periventricular white matter damage [12–15]. In the rat, postnatal day (P) 1–5 is the preterm equivalent of 23–32 weeks human gestation [16,17]. At these times, late oligodendrocyte (OL) progenitors (a.k.a., premyelinating OLs; pre-OLs), which are highly vulnerable to oxidative injury, are present in great numbers [18]. Thus, the study of microbleeds in the neonatal rat brain at full gestation may give insights into the consequences of microbleeds in human preterm infants during critical periods of white matter development.

Previous work from this laboratory showed that transient intrauterine ischemia (IUI) on embryonic day (E) 19 induces an angiogenic response in the rat brain that is characterized by upregulation of matrix metalloproteinase 9 (MMP9) and loss of microvascular basement membrane collagen IV [19–21]. The loss of collagen IV renders microvessels susceptible to rupture, such that an increase in venous pressure induced after birth results in cerebral microbleeds [19–21].

Apart from IUI, another potent pro-angiogenic stimulus is lipopolysaccharide (LPS), the canonical Toll-like receptor 4 ligand [22]. We hypothesized that the combined pro-angiogenic insults of IUI plus LPS would lead to a robust angiogenic response that would be associated with a strong proclivity to perinatal cerebral microbleeds. Here, we report on a novel rat model with spontaneous cerebral microbleeds sustained during birth. Rat fetuses were subjected to the combined pro-angiogenic stimuli of IUI plus low-dose maternal LPS (mLPS) on E19. Each insult alone was well tolerated, but the combination of IUI and mLPS was surprisingly harmful–in pups born naturally at E21-22 by vaginal delivery, spontaneous cerebral microbleeds were common, were associated with severe white matter and axonal abnormalities, and with prominent motor and behavioral deficits that persisted well into adolescence. Notably, the severity of cerebral microbleeds could be reduced by abdominal delivery, resulting in better preservation of white matter and axons, and better neurological function. Our findings underscore the great potential harm of cerebral microbleeds experienced when pre-OLs dominate.

## Materials and methods

### Ethics statement, and care and use of animals

We certify that all applicable institutional and governmental regulations concerning the ethical use of animals were followed during the course of this research. Animal experiments were performed under a protocol approved by the Institutional Animal Care and Use Committee of the University of Maryland, Baltimore and in accordance with the relevant guidelines and regulations as stipulated in the United States National Institutes of Health Guide for the Care and Use of Laboratory Animals. The University of Maryland School of Medicine Veterinary Resources Program is fully accredited by the American Association for the Accreditation of Laboratory Animal Care. The program of animal care is directed by full-time specialty-trained, laboratory animal veterinarians. All efforts were made to minimize the number of animals used and their suffering.

Timed-pregnant Wistar dams were purchased from Harlan Laboratories (Indianapolis, Ind.) and shipped to arrive between embryonic day (E) 14–16. E1 corresponds to the day of sperm-positivity following overnight mating. Pregnant dams were housed in individual cages with a 12-hour light-dark cycle. They were fed chow (2018 Teklad Global 18% protein rodent diet; Envigo, Frederick, MD) and water *ad libitum*. A wood enrichment block (4 × 4 cm) was placed in each cage. During the time leading up to parturition, care was taken to closely monitor but not overly disturb the animals. The physical condition of the dams was checked twice daily until E19, at which time some of them were subjected to IUI (see below). Pre- and post-operatively, dams were monitored for signs of distress including but not limited to: trembling, vocalization, changes in normal activity, changes in urine/fecal matter. Immediately after completion of surgery, dams were monitored every 20 minutes for 2 hours, then at 1-hour intervals for 6 hours, then at 12-hour intervals until parturition. Dams also were monitored for normal pre-labor and nesting behavior.

After parturition, pups that were destined for post-natal behavioral analyses (see below) remained housed with their mothers until post-natal day (P) 21. At P21, pups were weaned and separated into cages based on sex and were maintained using the same protocol as above.

As relates to surgery, all efforts were made to minimize pain, distress and surgical complications. Survival surgery was carried out under aseptic conditions on dams anesthetized to a surgical level with 3–4% isoflurane in a mixture of N_2_O/O_2_, 70%/30%, after which anesthesia was maintained with 1.5–2% isoflurane via a nosecone. Adequacy of anesthesia was assessed frequently by lack of a withdrawal response to deep toe pinch. Core temperature was maintained at 37°C by placing the dam on a clean cloth overlying a servo-controlled heating pad controlled by a rectal probe. Topical eye ointment was administered to protect the cornea during anesthesia. Surgical procedures were performed in a sterile environment with aseptic technique to reduce the risk of infection. Surgical instruments were autoclaved for 20 minutes at 121°C. Oxygen saturation, respiratory rate, and heart rate were monitored using a Pulse Oximeter. After surgery, rats were placed in pre-warmed cages to maintain a core body temperature of 37°C.

Generally, rats awoke from anesthesia rapidly (<10 minutes) and showed minimal pain or distress. The analgesic, Buprenorphine, was administered subcutaneously (0.3–0.5 mg/kg) every 12 hours, as needed.

A preapproved protocol was in place for the early euthanasia of animals. If discomfort were to persist after surgery, or if an animal were to become severely ill or moribund, a veterinarian (TC) would advised on proper pain management or on whether the animal should be euthanized prior to the end of the experiment. The method of euthanasia consisted of an overdose of sodium pentobarbital (>100 mg/kg). The heart rate was monitored until asystole was detected, after which the animal was exsanguinated by transcardial puncture.

### Dual prenatal pro-angiogenic stimuli of IUI plus maternal LPS

Timed-pregnant dams underwent laparotomy on E19 to induce 20-minute IUI, as detailed previously [19].

During the same surgery, the external jugular vein was isolated and catheterized. LPS (600 ng/hour; from *Escherichia coli* 0127:B8; #L4516, Sigma, St. Louis, MO) was infused into the external jugular vein via osmotic minipump (8.0 μL/hour × 24 hours; model 2001D; Alzet, Cupertino, CA) for 24 hours. Infusion of 600 ng/hr LPS yielded serum levels of 4.5±0.9 EU/mL LPS (n = 7 rats), measured 16 hours after start of infusion, using the Limulus amebocyte lysate assay (Thermo Fisher Scientific). These serum levels mimic levels of LPS reported in pregnant women with chronic indolent or subclinical infections (1.62±2.21; range: 0.38–15 EU/mL) [23].

Normally, spontaneous, unaided vaginal delivery occurred 2–3 days later (E21–22). In some cases, pups were delivered by Caesarian-section (C-section). In these cases, 2 pregnant dams with identical pregnancy timing (E19) were subjected to the same prenatal pro-angiogenic stimuli (IUI+mLPS) as above. When one of the 2 dams began to give birth vaginally, the other (C-section) dam was immediately euthanized by decapitation (anesthesia was avoided to prevent sedation of the newborns and rejection by the foster dam), and the pups were rapidly delivered abdominally, in <1.5 minutes from the time of decapitation. An experimenter immediately stimulated the pups to encourage breathing. The time between decapitation of the dam and spontaneous breathing of the last pup was <2 minutes. Pups from the first dam that were born vaginally were euthanized, and pups from the decapitated dam were placed with the surviving dam for fostering.

No mortality was experienced for dams undergoing the above protocol. We observed an overall mortality in pups of 18.5% after IUI+mLPS; mLPS alone led to 7.5% mortality.

### Experimental series and group allocations

In *series 1*, dams were subjected to IUI+mLPS (15 dams); fetal brain tissues 12 and 24 hours later (E20) were harvested to assess mRNA and protein markers of angiogenesis, and proteolytic activity (*in situ* zymography). Fetal brain tissues from pups of naïve (no intervention/uninjured) dams were collected on E20 as well (CTR; 4 dams). In *series 2*, dams were subjected to either: (i) control (no insult) with vaginal delivery (CTR-VD group; n = 3 dams); (ii) mLPS alone with vaginal delivery (mLPS-VD group; n = 2); (iii) IUI+mLPS with vaginal delivery (PS-VD group; n = 3 dams); or (iv) IUI+mLPS with abdominal delivery (PS-AD group; n = 2 dams); tissues from newborns at P0 were harvested to assess collagen IV or microbleeds. In *series 3*, dams were subjected to either: (i) control (no insult) with vaginal delivery (CTR-VD group; n = 3 dams); (ii) IUI+mLPS with vaginal delivery (PS-VD group; n = 3 dams); or (iii) IUI+mLPS with abdominal delivery (PS-AD group; n = 2 dams); pups were used to assess neurological function postnatally and in adolescence, and their brains were harvested at P52 to assess myelination and axonopathy.

An unbiased experimenter randomly assigned pregnant rats to undergo either surgery (IUI+mLPS) or to serve as a naïve control. When two dams underwent surgery, the one that did not begin vaginal labor first was allocated to the abdominal delivery group. In experimental *series 1*, pups were randomly assigned to be used for: (i) mRNA analysis, (ii) immunolabeling, (iii) *in situ* zymography. As such, the method of assigning experimental groups was unbiased.

### Brain hemorrhage analysis

Coronal sections at P0 were collected at either: (i) a single coronal plane (tissues from *experimental series 1*); or (ii) at 9 coronal planes spaced at 500 μm intervals spanning 4 mm (tissues from *experimental series 2*), starting at the rostral end of the lateral ventricles. Unstained sections were analyzed. Hemorrhages were identified in an automated, unbiased manner using custom software that employs a “support vector machine” (SVM) (MathWorks, Natick, MA) [24]. For each true color image (RGB), the green and blue channels were isolated and were used for analysis. Training data, consisting of blood-positive and blood-negative pixels, were hand-selected from a single digitized brain slice that contains extravasated blood, and were used to train a 2-dimensional linear SVM. Notably, the training data were linearly separable with no classification error. The trained SVM was used to classify each pixel in all brain slice images as either blood-positive or blood-negative, yielding total area occupied by blood.

### Neurobehavioral assessments

All behavioral tests were conducted by two separate scientists, one of whom was blinded to group identity; disagreements in scoring were resolved by consensus. Postnatal neurofunction was assessed using the *Righting Reflex* (at P3–P14) and *Negative Geotaxis* (at P3–P14) [25]. Adolescent social behavior was assessed using *Avoidance of the Center of an Open Field* (at P24) [26], *Avoidance of the Open Arms of the Elevated Plus Maze* (at P31) [26] and *Thigmotaxis during the First Trial in the Morris Water Maze* (at P35) [19,21]. Adolescent vestibulomotor function was assessed using *Rearing* (at P31) [27], *Beam Balance* (at P31) [19], and *Grip Strength* (at P31) [27] [19,21]. Adolescent spatial learning and memory were assessed using the Morris Water Maze for incremental spatial learning (at P35–39), memory probe (at P40), and rapid learning (at P42). The specific Morris Water Maze paradigms used in this laboratory have been described [19,21].

### RNA isolation and quantitative real-time polymerase chain reaction

Individual brains (n = 5) were used for analysis. Total RNA was isolated using Trizol Reagent (Invitrogen), and RNA was further purified by treatment with DNase I (Invitrogen). cDNA was synthesized from 2 μg of total RNA of each sample using SuperScript III Reverse Transcriptase (Invitrogen), and used for quantification of mRNA abundance of *Vegf*, *Cd31*, *Mmp2*, *Mmp9*, *Timp1*, *Timp2* and *Gapdh* by real-time PCR (ABI Prism 7300; Applied Biosystems, Carlsbad, CA). The primers used are shown in Table 1.

**Table 1:** qPCR primers used in this study.

Gene	Ref Seq	Forward (5’ to 3’)	Reverse (5’ to 3’)
Vegf	NM_031836	CTGCTGCAATGATGAAGCCCTG	GCTGCAGGAAGCTCATCTCTCC
Cd31	NM_031591	CCAAGGCCAGTAGCATCCTGGTC	GGATGGTGAAGTTAGCTACAGG
Mmp2	NM_031054	CAAGGACGGACTCCTGGCACAT	TACTCGCCATCAGCGTTCCCAT
Mmp9	NM_031055	CCAGTAGACAATCCTTGCAATGTG	CTCCGTGATTCGAGAACTTCCAATA
Timp1	NM_053819	TCCTGGTTCCCTGGCATAATCT	ATGACTGTCACTCTCCAGTTTGC
Timp2	NM_021989	GGCCAAAGCAGTGAGCGAGAAG	GCCGTGTAGATAAATTCGATGTC
Gapdh	NM_017008	CATCACTGCCACTCAGAAGACTG	ATGCCAGTGAGCTTCCCGTTCAG

### Histology and immunohistochemistry

The brains of E20 (for CD31, MMP2 and MMP9), P0 (for hematoxylin and eosin and collagen IV), or P52 (for Luxol Fast Blue, myelin basic protein and SMI-312) rats that had been perfused with 10% neutral buffered formalin were post-fixed 2–3 days in paraformaldehyde. Brains were transferred to 30% sucrose for cryopreservation then frozen in OCT. Cryosections (10 μm) were stained with hematoxylin and eosin (H&E) or Luxol fast blue (LFB) following standard protocols [28].

Immunohistochemistry was performed as described [20,28]. Sections were incubated overnight with primary antibodies, including: goat anti-CD31 (PECAM-1) (1:200, sc-1506; Santa Cruz Biotechnology, Santa Cruz, CA); rabbit anti-MMP2 (1:100; sc-10736; Santa Cruz); rabbit anti-MMP9 (1:200; #ab38898; Abcam, Cambridge, MA); rabbit anti-collagen IV (1:400; #ab6586; Abcam); rat anti-myelin basic protein (MBP) (1:200; #ab7349; Abcam); mouse anti-SMI-312 (1:200; #837901; BioLegend, San Diego, CA) at 4°C. After several rinses in PBS, sections were incubated with species-appropriate fluorescent secondary antibodies (Alexa Fluor 488 and 555, Molecular Probes, Invitrogen, Carlsbad, CA) for 1 hour at room temperature. Controls included the omission of primary antibodies.

### Quantification of specific labeling

Unbiased measurements of specific labeling within regions of interest (ROI) were obtained using NIS-Elements AR software (Nikon Instruments, Melville, NY) from sections immunolabeled or stained in a single batch. All images for a given signal were captured using uniform parameters of magnification, area, exposure, and gain. Segmentation analysis was performed by computing a histogram of pixel intensity for a particular ROI, and pixels were classified as having specific labeling, based on signal intensity greater than 2x that of background. The area occupied by pixels with specific labeling was used to determine the percent area in the ROI with specific labeling (% ROI).

For CD31, MMP2 and MMP9, the ROIs were a rectangle, 1500 x 500 μm, above the corpus callosum, plus an oval, 1000 x 500 μm, below the corpus callosum, plus a circle, 500 μm diameter, below the hippocampus. For collagen IV, the ROI was the entire coronal section (level 5; 2.0 mm behind the rostral extent of the lateral ventricle). For LFB, the ROI was a rectangle, 1000 x 500 μm, on the corpus callosum, in line with its long axis. For MBP, the ROI was a rectangle, 1500 x 500 μm, above the corpus callosum. For SMI-312, the ROI was a rectangle, 1500 x 500 μm, above the corpus callosum, plus an oval, 1000 x 500 μm, below the corpus callosum.

### Immunoblot

Individual brains (n = 3/group) were used for analysis. Proteins were extracted from formalin-fixed frozen brains harvested from the animals at P52 (*experimental series 3*). Rat brain tissue lysates were prepared from the dorsal half of 1-mm thick coronal sections adjacent to the sections used for immunohistochemistry. Protein extraction was performed using a Qproteome FFPE tissue kit (Qiagen, Valencia, CA), following the manufacturer’s instructions. Briefly, tissues (~100 mg) were homogenized in 300 μL of FFPE solution, incubated on ice for 5 minutes, following by incubation in a heating block incubator for 20 minutes at 100°C, and 120 minutes at 80°C. Insoluble aggregates were removed by centrifugation. To maximize protein yield, the pellet was resuspended in PBS containing 1% triton X100, incubated at room temperature for 10 minutes, and centrifuged again. Supernatants obtained after the first and second centrifugations were combined. The protein concentration was determined using a commercial protein assay solution (Bio-Rad, Hercules, CA). Equal amount of protein lysates were used for immunoblot analysis.

### In situ zymography

In situ zymography was performed on coronal sections from the brains harvested from the fetuses at E20, (*experimental series 1*). FITC-labeled DQ-gelatin (available in a gelatinase/collagenase assay kit, EnzChek, Thermo Fisher Scientific) was used as a substrate for degradation by gelatinases as described [29]. Brains were dissected on dry ice, quick-frozen with cold isopentane, and stored at −80°C until used. The brain blocks were cut into 12 μm sections using a cryostat (Leica, Wetzler, Germany). Mounted sections were thawed and incubated with reaction buffer (0.05 m Tris-HCl, 0.15 m NaCl, 5 mm CaCl_2_, and 0.2 mm NaN_3_, pH 7.6) containing 40 μg/ml DQ gelatin for 3 hours at 37°C. At the end of the incubation, sections were fixed for 10 minutes in 10% formalin and incubated for 2 hours at 37°C with anti-RECA-1 antibody (1:100; Therrmo Fisher Scientific) followed by 30 minutes incubation with AlexaFluor 555-conjugated secondary antibody (1:500; Therrmo Fisher Scientific). FITC enzymatic activity signal and RECA-1 immunolabeled blood vessels were visualized and photographed using fluorescence microscopy (Nikon Eclipse 90i).

### Statistics

Data are presented as mean±SE. Statistical analyses (ANOVA with post-hoc Tukey’s comparisons) were performed using Origin Pro (V8; OriginLab, North Hampton, MA).

## Results

### Angiogenic response

**Fig 1:**
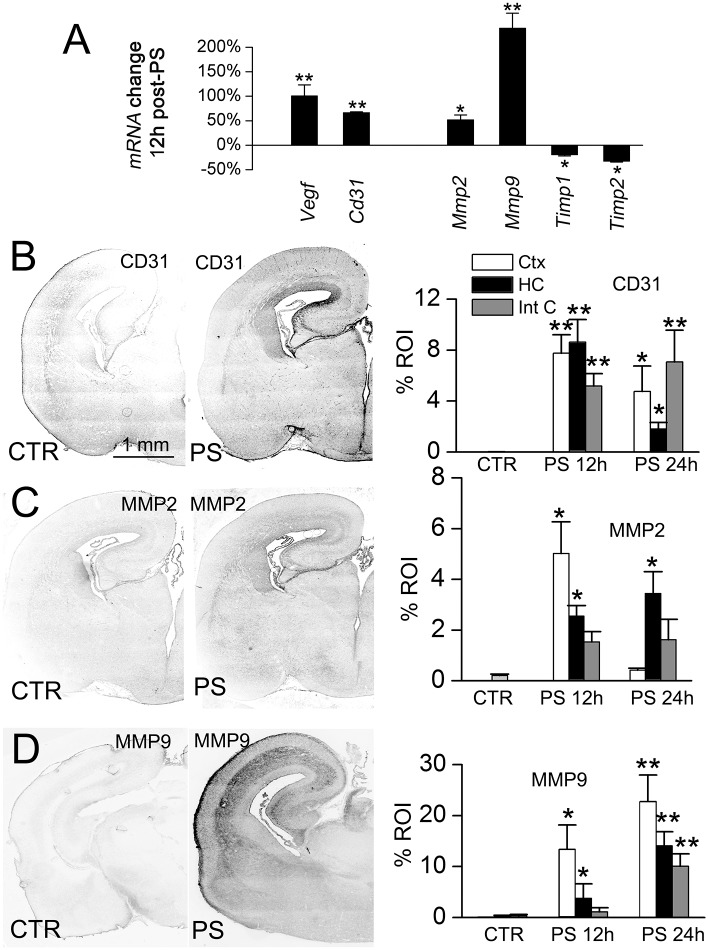
IUI+mLPS alters the expression of angiogenic markers. **A**: qPCR of mRNA for *Vegf*, *Cd31*, *Mmp2*, *Mmp9*, *Timp1* and *Timp2*, 12 hours after IUI+mLPS; data expressed as percent change with respect to naïve controls; 3 pups per group; *, *p*<0.05; **, *p*<0.01. **B–D**: Immunolabeling (*left*), with quantification (*right*), for the angiogenic markers, CD31, MMP2 and MMP9, on E20 in naïve controls (CTR) and 12 or 24 hours after dual prenatal pro-angiogenic stimuli (PS), in cortex (white bars), hippocampus (black bars) and internal capsule (gray bars); 3 pups per group.

**Fig 2:**
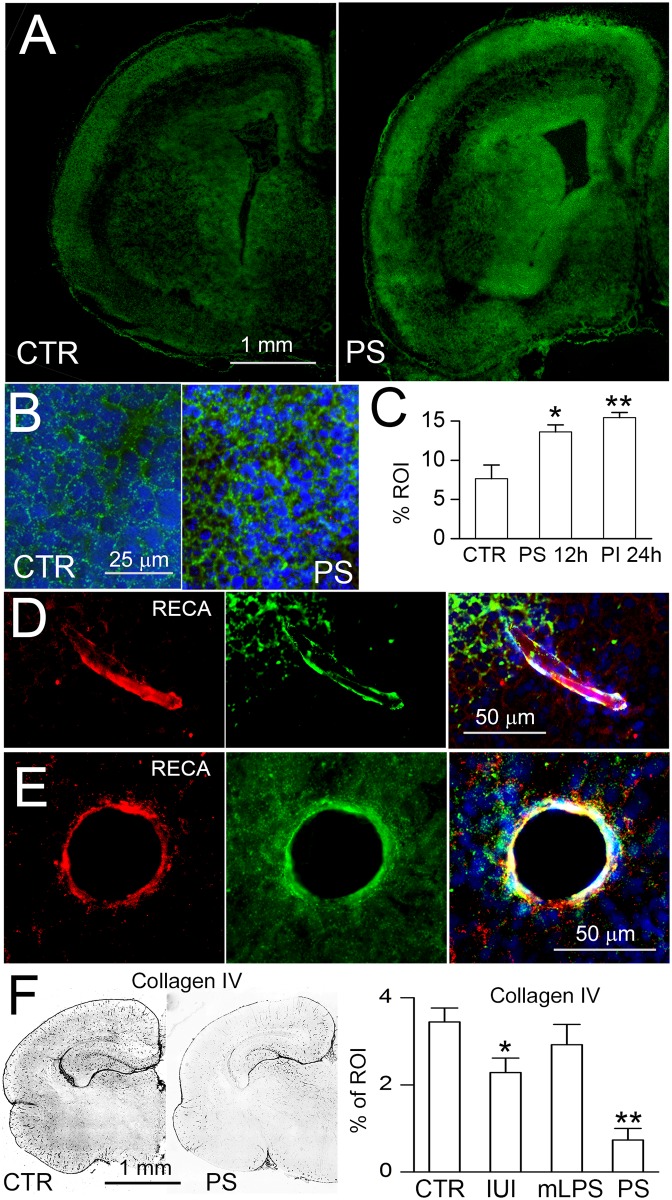
IUI+mLPS increases proteolytic activity and decreases collagen IV immunoreactivity. **A–C**: *In situ* zymography (A,B), with quantification (C), of coronal sections from naïve control (CTR) and 24 hours after dual prenatal pro-angiogenic stimuli (PS) of IUI+mLPS, shown at low (A) and at high (B) magnification; the subventricular zone is shown in (B); nuclei stained with DAPI (blue); scale bars, 1 mm (A), 25 μm (B); 3 pups per group; *, *p*<0.05; **, *p*<0.01. **D,E**: Images of vessels identified by immunolabeling for RECA (red), that show proteolytic activity on *in situ* zymography (green); merged images are shown on the right; nuclei stained with DAPI (blue); scale bars, 50 μm. **F**: Immunolabeling for collagen IV (*left*), with quantification (*right*), on P0 in naïve controls (CTR), after IUI alone, after mLPS alone, and after the dual pro-angiogenic stimuli of IUI+mLPS (PS), in all cases after vaginal delivery, in coronal brain sections; 5 pups per group; tissues from the IUI alone group were from a previous study [20].

Fetuses were exposed *in utero* to the dual pro-angiogenic stimuli of IUI plus low-dose maternal LPS (IUI+mLPS) on E19. Brains were studied 12–24 hours later (on E20) to evaluate markers of angiogenesis, including mRNA for *Vegf*, *cluster of differentiation 31 (Cd31)*, *Mmp2*, *Mmp9*, *tissue inhibitor of metalloproteinase (Timp) 1* and *Timp2*, as well as protein for CD31 (PECAM-1), MMP2 and MMP9 [30]. Quantitative real-time PCR showed that all of these angiogenic markers were significantly altered in periventricular areas of fetuses exposed to the dual stimuli, compared to naïve controls (Fig 1A). In accord with the mRNA findings, immunoreactivities for CD31, MMP2 and MMP9 were significantly elevated, although the temporal profiles in individual regions differed (Fig 1B–1D). *In situ* zymography showed a significant increase in proteolytic activity, including in the subventricular zone and in periventricular vessels (Fig 2A–2C), consistent with the increases in *Mmp2*/MMP2, *Mmp9*/MMP9, and the decreases in *Timp1* and *Timp2*.

Fetuses that had been exposed to various stimuli on E19 (IUI or mLPS or both) were studied on the day of birth (P0), to evaluate collagen IV. As previously reported, IUI alone reduced collagen IV immunoreactivity [20], whereas mLPS alone did not (Fig 2F). However, the angiogenic response to IUI+mLPS was associated with a marked reduction in immunoreactivity for microvascular collagen IV (Fig 2F).

### Periventricular hemorrhages

**Fig 3:**
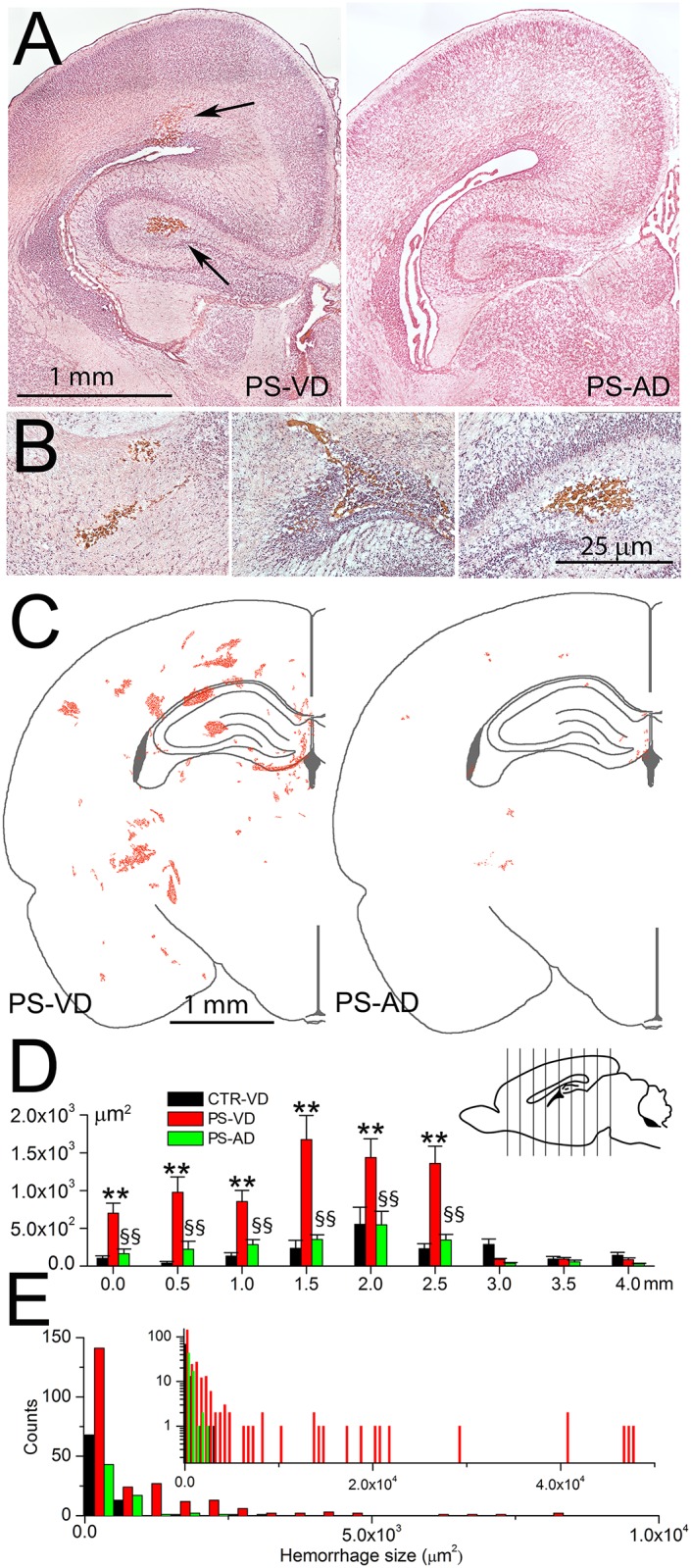
Cerebral microbleeds are linked to pro-angiogenic stimuli *in utero* followed by vaginal delivery. **A,B**: H&E-stained sections of P0 brains showing representative microbleeds (*arrows*) in pup following prenatal pro-angiogenic stimuli with vaginal delivery (PS-VD) (A, *left panel*; B, *all panels*) but not following prenatal pro-angiogenic stimuli with abdominal delivery (PS-AD) (A, *right panel*); scale bars, 1 and 0.25 mm in (A) and (B), respectively. **C**: Maps showing the locations and sizes of microbleeds identified in the coronal section 2.5 mm from the rostral extent of the lateral ventricle, on P0 in PS-VD pups (*left panel*) and in PS-AD pups (*right panel*); data from the right and left hemispheres of 10 pups are superimposed; scale bar, 1 mm. **D**: Averages of the total area occupied by hemorrhages in 9 coronal sections (see *inset*) in CTR-VD pups, PS-VD pups, and in PS-AD pups; 10 pups per group; **, *p*<0.01 comparing CTR-VD and PS-VD; §§, *p*<0.01 comparing PS-VD and PS-AD. **E**: Histogram showing the frequency distribution of microbleeds by size in the 3 groups; same coronal plane as in (C); inset shows the data plotted with an extended abscissa and the ordinate in log scale.

Pups exposed *in utero* to IUI+mLPS on E19 that were born naturally at E21–22 by vaginal delivery exhibited a high incidence of spontaneous cerebral microbleeds (Fig 3). On P0, multiple microbleeds were identified in all pups. Microbleeds were located predominantly in periventricular areas, which are vascular watershed territories [31].

Exposure to the dual stimuli of IUI+mLPS at E19 was required to produce significant microbleeds. Pups exposed to IUI alone at E19 and born vaginally at E21-22 exhibit minimal hemorrhages on P0 [19]. Here, comparing the total area of hemorrhage at a single coronal plane 2.5 mm behind the rostral tip of the lateral ventricles (*experimental series 1*, see Methods) gave values of 270±76, 287±73 and 2,744±365 μm^2^, in naïve pups with vaginal delivery, in pups exposed to LPS alone at E19 with vaginal delivery, and in pups with the dual stimuli of IUI+mLPS at E19 with vaginal delivery (Fig 3C). Thus, exposure to the dual pro-angiogenic stimuli was associated with a 10-fold increase in hemorrhages compared to naïve and LPS alone.

In pups exposed *in utero* to the same dual pro-angiogenic stimuli of IUI+mLPS on E19, but that were delivered by C-section on E21, microbleeds were significantly reduced compared to vaginally delivered pups (Fig 3C).

Hemorrhages were quantified at multiple coronal planes in three groups: naïve control pups (no prenatal insult) with Vaginal Delivery (CTR-VD), pups following dual Pro-angiogenic Stimuli (IUI+mLPS) with Vaginal Delivery (PS-VD), and pups following dual Pro-angiogenic Stimuli with Abdominal Delivery (PS-AD) (*experimental series 2*, see Methods). Microbleeds were identified in all 3 groups, in all coronal planes throughout the cerebrum (Fig 3D). However, in each coronal plane, the total area occupied by hemorrhages was significantly greater in PS-VD pups compared to PS-AD or CTR-VD pups (Fig 3D). The total areas occupied by hemorrhages in the CTR-VD and PS-AD groups were not significantly different.

Data from a representative coronal plane (2.5 mm behind the rostral tip of the lateral ventricles) in these 3 groups were analyzed further. Average microbleed sizes were 269±56, 3097±652 and 340±56 μm^2^ (corresponding to diameters of 19, 63, 21 μm) in the 3 groups, respectively. The smallest, most frequent hemorrhages (0–500 μm^2^), which were present in all 3 groups, were twice as frequent in the PS-VD group compared to the PS-AD and CTR-VD groups (Fig 3E). Larger hemorrhages were present only in the PS-VD group (Fig 3E).

### Neurological function

**Fig 4:**
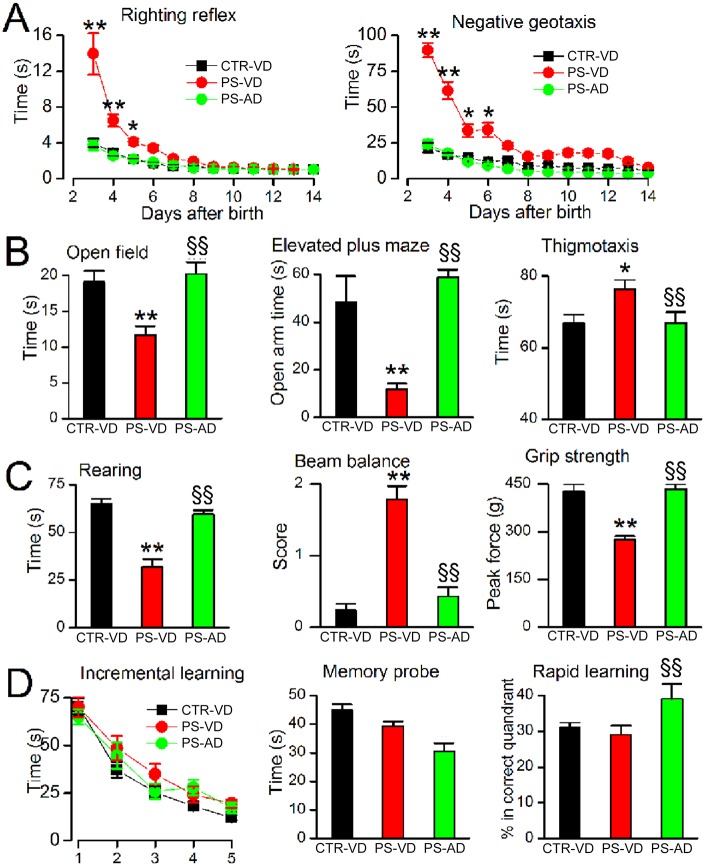
The effect of cerebral microbleeds on neurological function. **A**: Performance on the righting reflex and on the negative geotaxis test on P3–14 in naïve pups with vaginal delivery (CTR-VD), pups following prenatal pro-angiogenic stimuli with vaginal delivery (PS-VD), and pups following prenatal pro-angiogenic stimuli with abdominal delivery (PS-AD). **B**: Performance on the open field test at P24, the elevated plus maze at P31, and on thigmotaxis at P35, in CTR-VD pups, PS-VD pups, and PS-AD pups. **C**: Spontaneous rearing at P31, performance on the beam balance test at P31, and grip strength at P31 in CTR-VD pups, PS-VD pups, and PS-AD pups. **D**: Incremental spatial learning on P35–39, performance on the memory probe at P40, and on the rapid learning test at P42 in CTR-VD pups, PS-VD pups, and PS-AD pups. For all panels, 19–25 pups/group; * and **, *p*<0.05 and 0.01, respectively, comparing CTR-VD and PS-VD; §§, *p*< 0.01comparing PS-VD and PS-AD.

On P3–14, PS-VD pups exhibited severe abnormalities on the righting reflex and negative geotaxis (Fig 4A). By contrast, PS-AD pups performed similarly to controls (Fig 4A).

In adolescence (P24–35), PS-VD rats displayed an anxiety-like phenotype, with avoidance of the center of the open field chamber, avoidance of the open arms of the elevated plus maze, and increased time in the thigmotaxis zone during the first trial in the Morris water maze (Fig 4B). By contrast, PS-AD rats showed minimal anxiety-like behaviors that were no different than controls (Fig 4B).

In adolescence (P24–31), PS-VD rats displayed abnormal performance on rearing, beam balance, and grip strength (Fig 4C). By contrast, in PS-AD rats, these functions were no different than controls (Fig 4C).

In adolescent rats (P35–45) exposed prenatally to IUI+mLPS, incremental spatial learning was not significantly affected, and spatial memory was inconsistently affected by vaginal versus abdominal delivery (Fig 4D).

### White matter and axons

**Fig 5:**
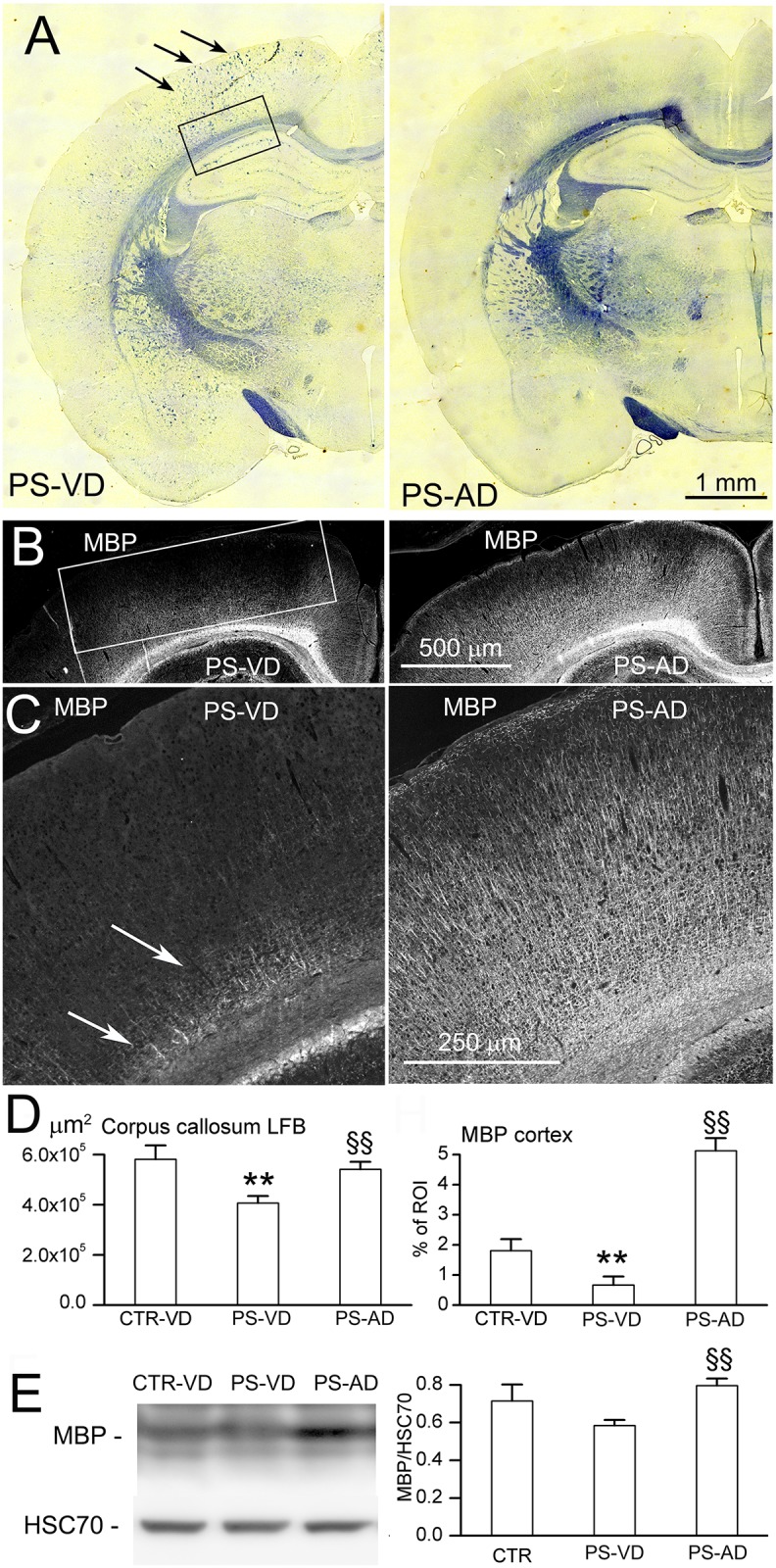
The effect of cerebral microbleeds on myelination. **A–D**: Representative images (A–C), with quantification (D), of LFB staining (A) and MBP immunolabeling (B,C) at P52 in naïve pups with vaginal delivery (CTR-VD), pups following prenatal pro-angiogenic stimuli with vaginal delivery (PS-VD), and in pups following prenatal pro-angiogenic stimuli with abdominal delivery (PS-AD); arrows in (A) point to clumped myelinated fibers; arrows in (C) point to poorly myelinated fibers above corpus callosum; rectangles show ROI’s that were quantified; 7 rats/group; **, *p*<0.01 comparing CTR-VD and PS-VD; §§, *p*<0.01 comparing PS-VD and PS-AD; bars, 1 mm (A), 500 μm (B), 250 μm (C). **E**: Immunoblot (*left*), with quantification (*right*), of all bands of MBP at P52 in CTR-VD, PS-VD, and PS-AD rats; HSC70 used as loading control; 3 rats per group.

White matter and axonal abnormalities were evaluated at P52. PS-VD rats exhibited severe white matter abnormalities. Compared to controls, Luxol Fast Blue staining and myelin basic protein immunolabeling and immunoblot revealed hypomyelination of the corpus callosum and cortex, along with clumped myelinated fibers [32] in both the cortex and hippocampus (Fig 5). Compared to PS-VD rats, PS-AD rats showed significantly better myelination and only rare clumped myelinated fibers (Fig 5).

**Fig 6:**
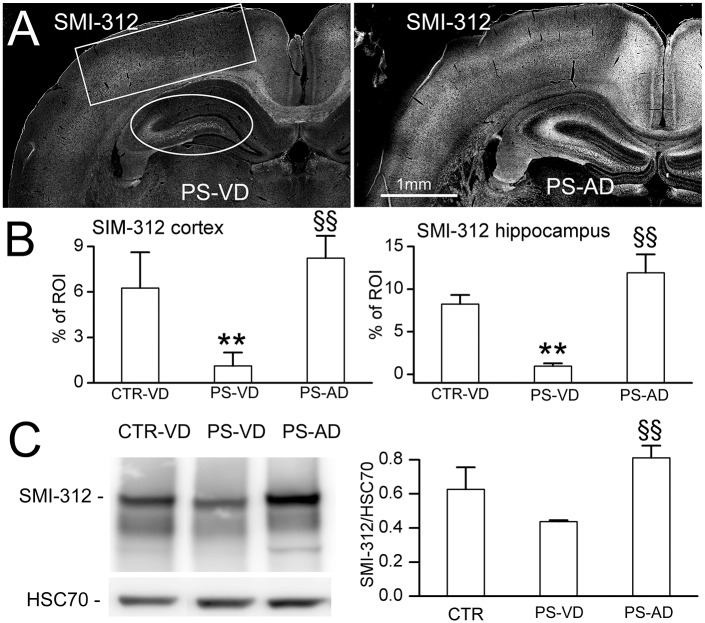
The effect of cerebral microbleeds on axonal development. **A,B**: Representative images (A), with quantification (B), of immunolabeling for SIM-312 at P52 in naïve pups with vaginal delivery (CTR-VD), pups following prenatal pro-angiogenic stimuli with vaginal delivery (PS-VD), and in pups following prenatal pro-angiogenic stimuli with abdominal delivery (PS-AD); rectangle and oval show regions of interest (ROI) that were quantified; 7 rats/group; **, *p*<0.01 comparing CTR-VD and PS-VD; §§, *p*<0.01 comparing PS-VD and PS-AD; bar, 500 μm (A). **C**: Immunoblot (*left*), with quantification (*right*), of SIM-312 at P52 in CTR-VD, PS-VD, and PS-AD rats; HSC70 used as loading control; 3 rats per group.

PS-VD rats also exhibited severe axonal abnormalities. Compared to controls, SMI-312 immunolabeling and immunoblot revealed severe axonopathy involving cortex and hippocampus (Fig 6). Compared to PS-VD rats, PS-AD rats showed significantly better axonal development (Fig 6).

## Discussion

The principal findings of the present study are that, in a rat model: (i) an angiogenenic response induced shortly before birth can predispose to cerebral microbleeds during birth; (ii) microbleeds can damage white matter, especially if they occur during the stage of white matter development dominated by pre-OLs; (iii) in rats, white matter damage induced by microbleeds sustained at birth is accompanied by severe axonopathy and severe adolescent neurological dysfunction, reminiscent of periventricular leukomalacia in humans; (iv) when microbleeds in susceptible animals are reduced, e.g., by abdominal delivery, significantly better myelination, axonal preservation, and neurological function are observed, regardless of potentially harmful prenatal exposures.

Factors known to predispose to perinatal brain hemorrhages are collagen IV maldevelopment [33] and collagen IV deficiency that can be due to either constitutive [34] or induced angiogenesis [20]. In this context, it is important to distinguish between full-blown, active angiogenesis, as occurs in the preterm germinal matrix, and the initiation of an angiogenic response, which may follow a brief, transient pro-angiogenic stimulus such as hypoxia. The former results in the active formation of new microvasculature, whereas the latter suggests that the angiogenic process may have been initiated but not completed. In our model, new microvessels were not observed, presumably because the prenatal pro-angiogenic stimuli were not sustained. However, we did observe a robust angiogenenic response that was characterized by upregulation of VEGF, CD31, MMP2 and MMP9, downregulation of TIMP1 and TIMP2 [30], augmented MMP proteolytic activity, and degradation of microvascular collagen IV and laminin [20]. Similar findings involving MMPs and TIMPs have been reported in animal models and in humans following perinatal ischemic insults [35–39]. The early phases of the angiogenic response that involve matrix degradation appear to be sufficient to leave existing microvessels structurally weakened and susceptible to subsequent mechanical perturbations.

We previously reported that 20-minute IUI and its angiogenic response in the rat were relatively harmless unless newborn pups (P0) also were exposed to a second insult that caused an increase in venous pressure, in which case they suffered microbleeds with an anatomical distribution similar to that observed here and that were accompanied by lasting neurological abnormalities [19–21]. Apart from IUI, another potent angiogenic stimulus is LPS [22]. Here, we found that when IUI was followed by low-dose maternal LPS, the fetal angiogenic response was robust, such that natural vaginal delivery by itself was sufficient to induce microbleeds. In the rat, uterine contractions that expel the fetus at birth are associated with periodic rises in intrauterine pressure that peak at 30 mm Hg [40], the same pressure recorded in the jugular vein associated with microbleeds after IUI [19,21]. Periodic uterine contractions compress the fetus, and thus induce periodic venous hypertension during vaginal delivery, which we speculate may have been the immediate cause of the microbleeds, since avoiding vaginal delivery greatly reduced cerebral microbleeds.

The susceptibility to white matter injury during development coincides closely with the timing of the appearance of pre-OLs [18]. Pre-OLs *in vitro* are markedly more susceptible than mature OLs to free radical-mediated injury [41]. Redox-active iron, a catalyst in the production of hydroxyl radicals via the Fenton reaction, is a key participant in reactive oxygen species (ROS)-induced tissue injury and inflammation [42]. Excess periventricular iron, which can arise from hypoxia/ischemia (H/I), has been strongly linked to pre-OL apoptosis and periventricular white matter damage [14]. When pre-OLs are present in great numbers (in humans, 23–32 weeks postconception; in rats, P1–5), they are highly susceptible to oxidative injury, leading to early death or arrested maturation, resulting later in reduced numbers of mature, MBP-expressing OLs [43,44]. Notably, much of the previous work on this subject focused on pre-OL injury induced by one or both of the two classical insults, hypoxia/ischemia and LPS, applied during the critical window of vulnerability [45].

To our knowledge, the work reported here is the first to directly implicate cerebral microbleeds as a major contributor to severe pre-OL injury. We show that microbleeds sustained in the term rat, when pre-OLs dominate, contribute strongly to periventricular white matter damage, consistent with the known, potent pro-oxidant effect of extravasated blood products [46]. Moreover, haptoglobin, which is crucial for scavenging blood breakdown products [10], is largely absent at birth [47], compounding the insult by allowing blood breakdown products protracted opportunity to induce oxidative damage. Our findings suggest that microbleeds superimpose on the innate immune response to IUI+mLPS [48], and have an unexpected, disproportionately large impact on white matter development and on neurofunctional outcome. Even in the face of prenatal IUI+mLPS, if microbleeds were prevented, white matter was much better preserved and neurological function was near normal, whereas with microbleeds, white matter damage was prominent and neurological function was very abnormal.

In human neonates, intra- and peri-ventricular macrohemorrhages may be due to structurally weak vessels in the germinal matrix. These macrohemorrhages are quite different from the microbleeds in the present model. The larger hemorrhages that historically have been detected by cranial ultrasound are associated with major disabilities, such as cerebral palsy. With the advent of MRI, more subtle noncystic white matter injury, microbleeds (punctate hemorrhages), and cerebellar lesions, are increasingly diagnosed that are associated with cognitive and behavioral problems, the most prevalent and vexing issues among survivors of preterm birth [49]. To the extent that the etiology and the neurobiology of microbleeds are similar across species, our findings on microbleeds may be applicable to human preterm neonates who experience proangiogenic insults and cerebral microbleeds when pre-OLs dominate.

In humans, the question whether abdominal delivery affords neuroprotection is considered to be largely settled. Under some circumstances, abdominal delivery has been reported to protect neonates from brain macrohemorrhages [50–52] and possibly from leukomalacia [53]. However, the majority of reports on this topic indicate that the mode of birth does not influence neonatal outcome or the likelihood of cerebral palsy [54,55].

This study has limitations. While rats at term are developmentally or ontogenically similar to humans at the gestational age of 24–26 weeks, in the model, the rats are delivered at term, and so translating our findings to neonates requires caution. In our work developing this model, we did not separately analyze males and females, although sex is known to be an important factor in perinatal brain injury. Despite these and other limitations, the study of microbleeds in the neonatal rat brain at full gestation may give insights into the consequences of microbleeds in human preterm infants during critical periods of white matter development.

In conclusion, we show in a rat model that pro-angiogenic stimuli *in utero* can predispose to vascular fragility and lead to cerebral microbleeds that can have lasting damaging consequences on white matter development and neurological function.

## Supporting information

S1 File Original data. This file contains the original data quantified in the figures.
(XLSX)
