# Correction: Cerebral microbleeds in a neonatal rat model

**DOI:** 10.1371/journal.pone.0201831

**Published:** 2018-07-31

**Authors:** Brianna C. Theriault, Seung Kyoon Woo, Jason K. Karimy, Kaspar Keledjian, Jesse A. Stokum, Amrita Sarkar, Turhan Coksaygan, Svetlana Ivanova, Volodymyr Gerzanich, J. Marc Simard

The first author's last name is entered incorrectly. The correct name is: Brianna C. Theriault. The correct citation is: Theriault BC, Woo SK, Karimy JK, Keledjian K, Stokum JA, Sarkar A, et al. (2017) Cerebral microbleeds in a neonatal rat model. PLoS ONE 12(2): e0171163. https://doi.org/10.1371/journal.pone.0171163
